# Flexibility and thermal dynamic stability increase of dsDNA induced by Ru(bpy)_2_dppz^2+^ based on AFM and HRM technique

**DOI:** 10.1186/s13065-019-0584-9

**Published:** 2019-05-17

**Authors:** Fuchao Jia, Pascal Hébraud, Kezhen Han, Jing Wang, Xingguo Liang, Bo Liu

**Affiliations:** 1 Laboratory of Functional Molecules and Materials, School of Physics and Optoelectronic Engineering, Shandong University of Technology, Zibo, 255000 China; 2 Institut de Physique et Chimie des Matériaux de Strasbourg/Centre National de la Recherche Scientifique, University of Strasbourg, 67034 Strasbourg, France; 3 College of Food Science and Engineering, Ocean University of China, Qingdao, 266003 China

**Keywords:** DNA flexibility, Thermal dynamic stability, Ruthenium complex, Atomic force microscopy

## Abstract

Ru(bpy)_2_dppz^2+^ has been widely used as a probe for exploring the structure of double-stranded DNA (dsDNA). The flexibility change of DNA helix is important in many of its biological functions but not well understood. Here, flexibility change of dsDNA helix caused by intercalation with Ru(bpy)_2_dppz^2+^ was investigated using the atomic force microscopy. At first, the interactions between ruthenium complex and dsDNA helix were characterized and the binding site size (p = 2.87 bp) and binding constant (K_a_ = 5.9 * 10^7^ M^−1^) were determined by the relative extension of DNA helix using the equation of McGhee and von Hippel. By measuring intercalator-induced DNA elongation and the mean square of end-to-end distance at different molar ratios of Ru(bpy)_2_dppz^2+^ to dsDNA, the changes of persistence length under different ruthenium concentrations were determined by the worm-like chain model. We found that the persistence length of dsDNA decreased with increasing Ru(bpy)_2_dppz^2+^ concentration, demonstrating that the flexibility of dsDNA obviously enhanced due to the intercalation. Especially, the persistence length changed greatly from 54 to 34 nm on changing the molar ratio of ruthenium to dsDNA from 0 to 0.2. We speculated that the intercalation of dsDNA with Ru(bpy)_2_dppz^2+^ resulted in local deformation or bending of the DNA duplex. In addition, the thermal dynamic stability of DNA helix was measured with high resolution melting method which revealed the increase in thermal dynamic stability of DNA helix due to the ruthenium intercalation.

**Electronic supplementary material:**

The online version of this article (10.1186/s13065-019-0584-9) contains supplementary material, which is available to authorized users.

## Introduction

**Fig. 1:**
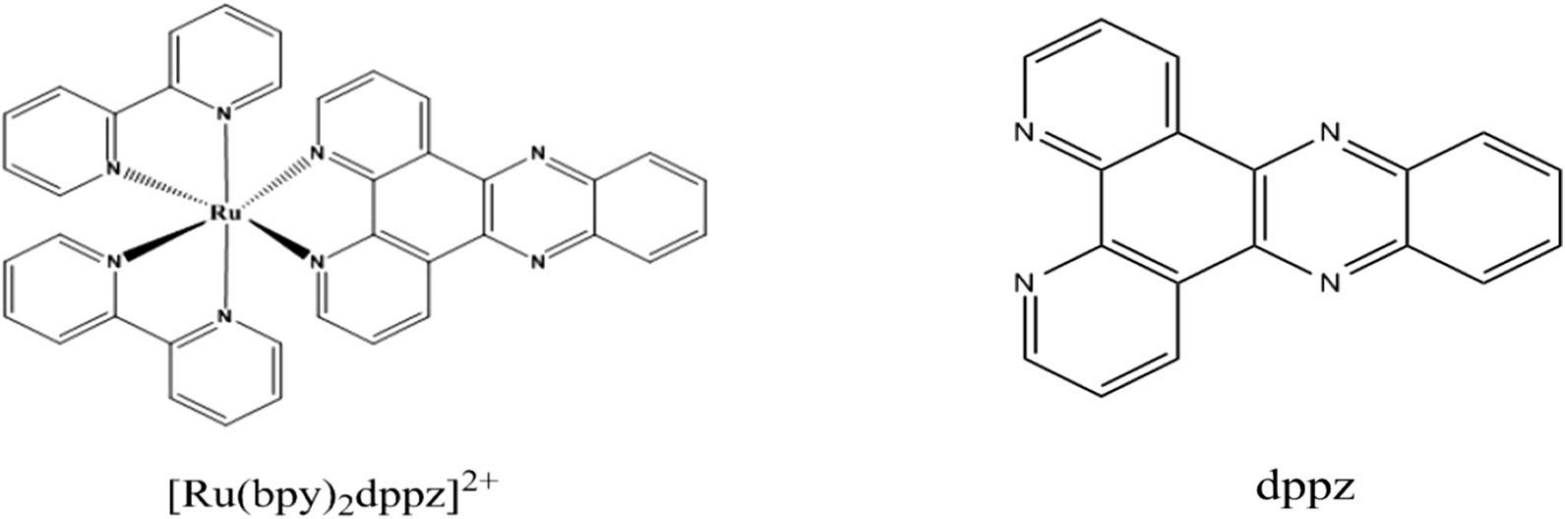
Chemical structure of ruthenium complex

Ruthenium complexes have been widely used in “molecular light switch”, deoxyribose nucleic acid (DNA) structure probes and anticancer drugs due to their good photochemical and photophysical properties [1–5]. The interactions between ruthenium complexes and DNA have been extensively studied since after the first observation of the “molecular light switch” effect of Ru(bpy)_2_dppz^2+^ (chemical structure is shown in Fig. 1) for DNA molecules [6–13]. The interaction between them can be regarded as the modification of double-strand DNA (dsDNA) which influence mainly on two aspects: (1) mechanical properties of DNA double strands which often characterized with the persistence length (it is a measure of how far a polymeric chain persists in a given direction), and (2) thermodynamic stability of DNA double strands. These two kinds of properties have great impacts on its overall shape as well as on many of biological functions, such as chromosomal DNA packaging, DNA damage repair, regulation of gene expression, and protein–DNA binding [14–18]. It is crucial to understand the change of biophysical properties of dsDNA induced by the ruthenium intercalation, which would be very helpful to interpret intercalate interference with biochemical processes.

Many different techniques have been used to determine the interaction between ruthenium and dsDNA [7, 9, 13, 19–21]. Single molecule stretching experiments and atomic force microscopy (AFM) are complementary techniques for measuring the interaction between metal complexes and dsDNA [21, 22]. The binding mode and binding constant can be determined by the single molecule stretching measurements, but normally these are force-dependent at non-equilibrium state. AFM is a powerful technique to study the biophysical properties of single DNA molecules, including DNA flexibility, and it provides the advantage of direct observation of the DNA molecules when adsorbed onto supporting surfaces [23–27]. Several AFM studies have evaluated the DNA persistence length and the conformational state of the nucleic acid polymer confined to an imaging plane [25, 28, 29]. However, few studies have reported the physical property change of DNA duplex induced by the Ru(bpy)_2_dppz^2+^ intercalation which could influence the biological process. In the present study, AFM was employed to characterize the change of conformation and contour length of dsDNA, and the variation of persistence length of dsDNA induced by ruthenium intercalation was calculated with worm-like chain model in two dimensions. The flexibility of dsDNA induced by the intercalation of Ru(bpy)_2_dppz^2+^ was obtained by calculating the persistence length change. Besides that, melting temperature (T_m_) measurements were performed with high resolution melting (HRM) method to understand the thermal dynamic stability of dsDNA after ruthenium intercalation. Understanding the changes in DNA helix induced by ruthenium intercalation will be very useful to design the novel and effective anti-cancer drugs.

## Materials and methods

### AFM sample preparation and imaging

Linearized plasmids (PBR322, 4361 base pairs) was purchased from Sangon Biotech and used without further purification. Ruthenium complex and dsDNA duplex were mixed in a buffer (10 mM Tris–HCl and 10 mM MgCl_2_, pH 7.0) with different molar ratios of ruthenium complex to dsDNA base pairs (0.1, 0.2, 0.33, 1, 2 and 3) whereas the final concentration of dsDNA was always maintained to be 0.5 ng/μL. 10 μL mixed solution was added onto the freshly cleaved mica and gently rinsed with 4 mL deionized water to remove extra divalent cations and gently blow-dried with nitrogen. The as-prepared sample was used for imaging at room temperature in air with Multimode IIIa AFM (Vecco Instruments, American). A series of 5 × 5 µm^2^ AFM images were captured under the tapping mode at 1 Hz scanning rate to avoid dragging of DNA by the tip. In our study, randomly curved and non-crossing DNA molecules were chosen and analyzed from many different images. Additionally, DNA molecule could be compressed under tapping mode AFM. In order to avoid the influence of tapping mode AFM, the measurements of relative length variance were performed, and the the length variance of different DNA molecule (pure DNA molecule is as control) were characterized under the same experimental conditions, so the tapping mode AFM had little influence on the experiments results.

### T_m_ measurement of dsDNA using HRM method

DNA oligonucleotides were synthesized and purified by iPAGE (Invitrogen, Shanghai, China). The ratios of ruthenium complex to dsDNA were maintained consistent for AFM experiments and measured the T_m_ with the well mixed solution. The temperature increased gradually from 35 to 99.5 °C and fluorescence data was collected with every 0.1 °C increment. All fluorescence data were acquired by at least two parallel tests, and the measurements of melting profiles were repeated at least twice. The specific informations are shown in supplementary materials.

## Results and discussion

### Measurement of flexibility change of dsDNA using AFM method

#### Tracing and image analysis

**Fig. 2:**
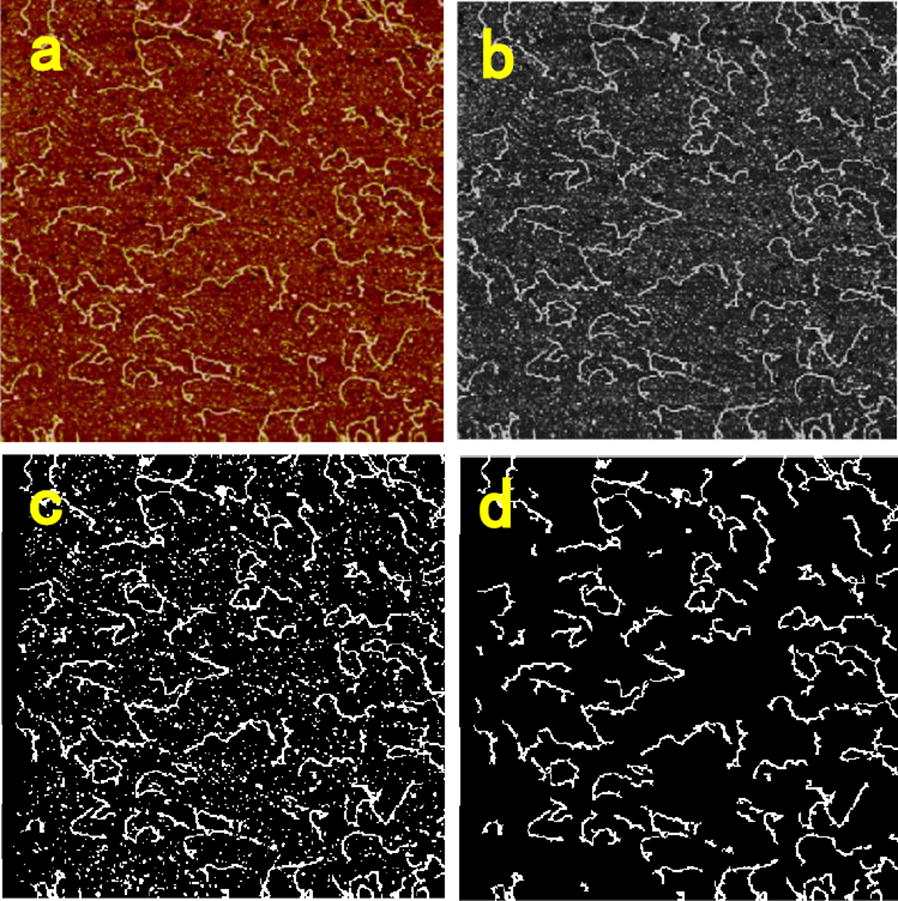
The process of dealing with raw image. **a** Raw image, **b** grayscale image, **c** binarized image, **d** noise reduction image

The obtained AFM images were analyzed by a custom unbiased program written with MATLAB. Briefly, the original image without background slope was converted into a binary image and randomly selected a pixel in each dsDNA and subsequently searched the extensions from the selected pixel to both sides till the ends. The distance between two pixels was determined by their position coordinates $\left( {\Delta {\text{l}} = \sqrt {(dx)^{2} + (dy)^{2} } } \right)$, and the contour length (the actual length of the polymeric chain from one end to another end along the polymer skeleton) of each dsDNA was obtained by summation of the distance $(\varSigma \Delta {\text{l)}}$ of all pixels on the DNA skeleton line. The process of dealing with the raw image is shown in Fig. 2.

#### Morphology and binding property analysis

**Fig. 3:**
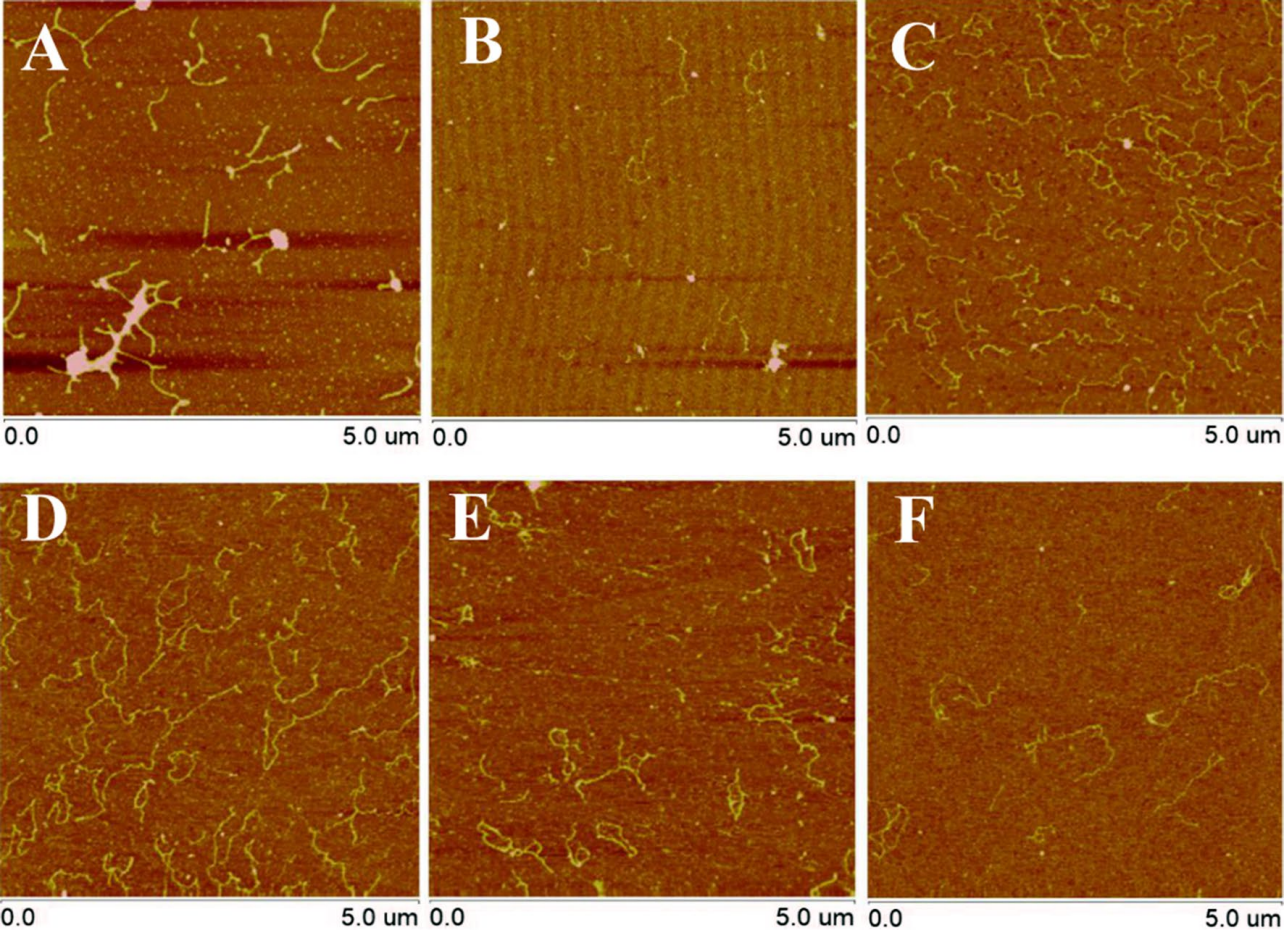
AFM images under different ratios of Ru/DNAbps (ratios; **A**: 0.1, **B**: 0.2, **C**: 0.33, **D**: 1, **E**: 2, **F**: 3)

**Fig. 4:**
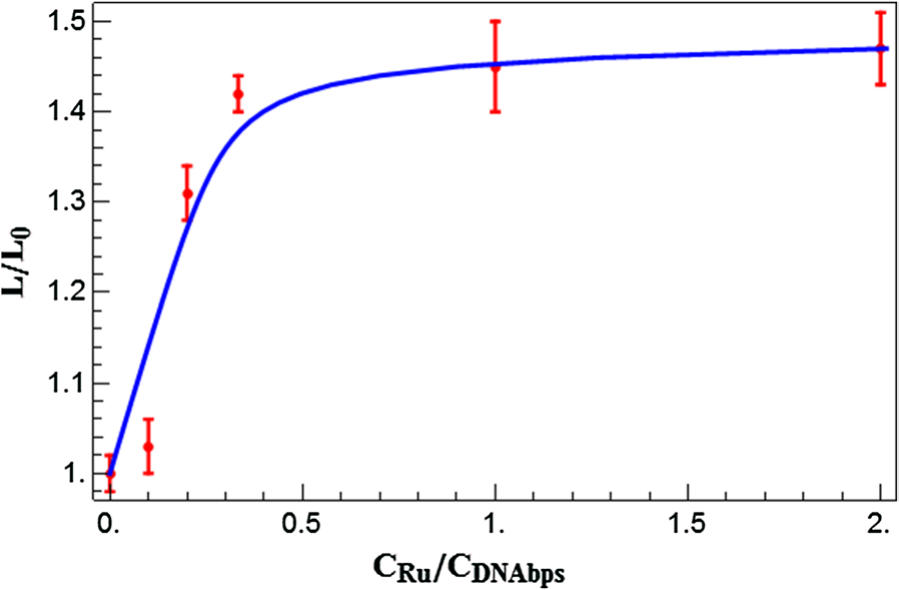
The change of DNA length as a function of the ruthenium intercalation. The curve presents the fractional extension (L/L_0_) defined as the increase of the end-to-end distance (L) due to the intercalation of Ru(bpy)_2_dppz^2+^ normalized by the natural length of the DNA molecule (without Ru), plotted over the Ru/DNAbps ratio (red circle). The blue solid line represents the fit from the model of McGhee and von Hippel (Eq. 1). The results were best fit for Ka: 5.9 * 10^6^ M^−1^ and p: 2.87 bp

In order to gain deeper insight into the influence of Ru(bpy)_2_dppz^2+^ intercalators on the conformational properties of dsDNA, it is critical to imaging individual DNA molecule on a mica surface under different ratios of Ru(bpy)_2_dppz^2+^ to DNA base pairs (Ru/DNAbps). Quantitative AFM images of dsDNA–ruthenium complexes on the mica surface were successfully and systematically captured in our study. In detail, the DNA fragments appeared like squiggly lines because of the resolution limit of the AFM (Fig. 3). Neither grooves nor helical twist could be resolved, but the overall shape could be seen and the length could be measured with reasonable accuracy. With increasing the ratio of Ru/DNAbps from 0.1 to 1, it was clearly observed that the contour length increased distinctly, which confirmed the intercalation of Ru(bpy)_2_dppz^2+^ and induced the local changes of dsDNA (Additional file 1). In Fig. 3A–D, dsDNA molecules showed very few crossings, whereas in Fig. 3E, F, dsDNA molecules were imaged in excess of Ru(bpy)_2_dppz^2+^ (ratio 2 and 3 of Ru/DNAbps) resulting in the condensation of many crossings. From these images, we can clearly observe the conformational change of dsDNA molecules with different ruthenium concentration. At saturated concentration, the dsDNA molecules appeared to be more condensed than compared with the low ratio of Ru/DNAbps, indicating that the dsDNA molecules was more flexible at high intercalator concentration than at low intercalator concentration which was consistent with the results of flexibility analysis. For determining the binding property between them, we used the converted equation of McGhee and von Hippel to obtain a relationship between the fractional extension L/L_0_ and the ratio of Ru/DNAbps (the plot is shown in Fig. 4) [30]. The obtained relation can be described as Eq. 1:

1 $$\frac{{C_{Ru} }}{{C_{{DNA{\text{bps}}}} }} = \nu + \frac{\nu }{{K_{a} (1 - p\nu )C_{{DNA{\text{bps}}}} }} \left [\frac{{1 - \left( {p - 1} \right)\nu }}{1 - p\nu }\right]^{p - 1}$$

2 $$\nu = \frac{{L - L_{0} }}{{L_{0} }}\frac{{\delta_{bp} }}{{\delta_{Ru} }}$$

where C_Ru_ and C_DNAbps_ are the concentration of total ruthenium and DNAbps, respectively. ν is the ratio between the bound intercalator and the total concentration of DNAbps, p is binding site size i.e., the number of base pair sites occluded by one bound Ru(bpy)_2_dppz^2+^ and K_a_ is the affinity constant. The relation between ν and contour length can be described with Eq. 2, where *L*_*0*_ is contour length without ruthenium intercalation, *L* is the contour length after its intercalation, $\delta_{bp}$ is the rise parameter per DNAbps of 0.34 nm and $\delta_{Ru}$ is the DNA elongation per bound one Ru(bpy)_2_dppz^2+^ of 0.5 nm.

It was feasible to derive the relative extension of DNA double stand as a function of ruthenium concentration per base pair concentration from the contour length measurement, and the results are plotted in Fig. 4. K_a_ of ruthenium complex and dsDNA interactions was estimated using the comparison between the experimental data and the equation converted from the theory developed by McGhee and von Hippel in the case of non-cooperative ligand binding [30]. Figure 4 shows the comparison between our experimental data and theoretical model. The best fitting data was obtained for the p and K_a_ values of 2.87 bp and 5.9 * 10^7^ M^−1^, respectively. These values are in good agreement with the values obtained by Williams et al. which was measured by stretching experiments with classical intercalator. In addition, as indicated by the plot of Fig. 4, the measured contour length of DNA increased with increasing Ru/DNAbps ratio. For the ratios of Ru/DNAbps is over 1, the observed contour length became saturated and the plateau indicated the 50% increase of the relative length. We speculated that ruthenium intercalation induced a change in the local structure of DNA helix resulting in a lengthening of the DNA strand. Which was consistent with previous results of classical intercalators [29, 31]. In cells, many DNA-distorting proteins used side chain intercalation to distort the DNA backbone which plays important roles for processing information in DNA and organizing chromosome DNA [32].

#### The flexibility of dsDNA analysis

In this part, we mainly presented the physical property of probed DNA molecules and how the binding of Ru(bpy)_2_dppz^2+^ affects the extension and the flexibility of DNA molecules. The flexibility of DNA can be characterized by its persistence length. Normally, there are two major methods to estimate the persistence length of adsorbed macro-molecules on the mica surface. The first method is based on the measurements of the end-to-end distance and the contour length which is very reliable when used on a large number of molecules [33]. The second method is based on the measurements of the angle between two small segments separated by a certain distance. This method is very sensitive to the local bending of the DNA molecules and does not require measurements of hundreds of molecules. However, AFM tip can easily lead to the local bending along the scanning direction. Herein, the first method was employed to measure the persistence length of dsDNA.

The straightforward approach is to consider the root mean square of end-to-end distance (⟨R^2^⟩) of an ensemble of identical polymers. Assuming the DNA to be at thermal equilibrium state, the 2D (two dimension) worm-like chain (WLC) model provides a relationship between the mean end to-end distance and contour length, and it can be described by the following equation [27]:

3 $$\left\langle {R^{2} } \right\rangle = 4L_{P} L_{C} \left( {1 - \frac{{2L_{P} }}{{L_{C} }} \left (1 - {\text{e}}^{{ - \frac{{{\text{L}}_{\text{C}} }}{{ 2L_{P} }}}} \right)} \right)$$

where *L*_*P*_ is the persistence length and *L*_*C*_ is the contour length. This nonlinear relationship needs to be inverted for *L*_*P*_ after measuring the mean-square end-to-end distance of DNA chain and the contour length, and the persistence length can be obtained by the Eq. 3. For longer chains ⟨R^2^⟩ ≈ 4L_P_L_C_, at least fifty DNA fragments were analyzed under every ratio in our study.

**Fig. 5:**
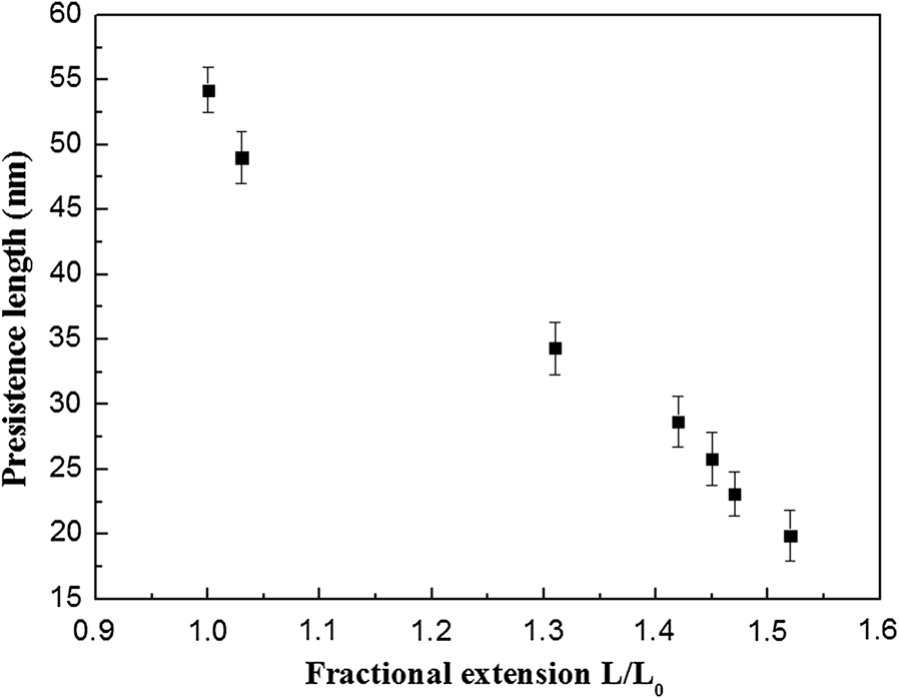
Dependence of persistence length Lp on fractional extension L/L0 the curve shows that Lp decreases with increasing Ru/DNAbps ratio

**Fig. 6:**
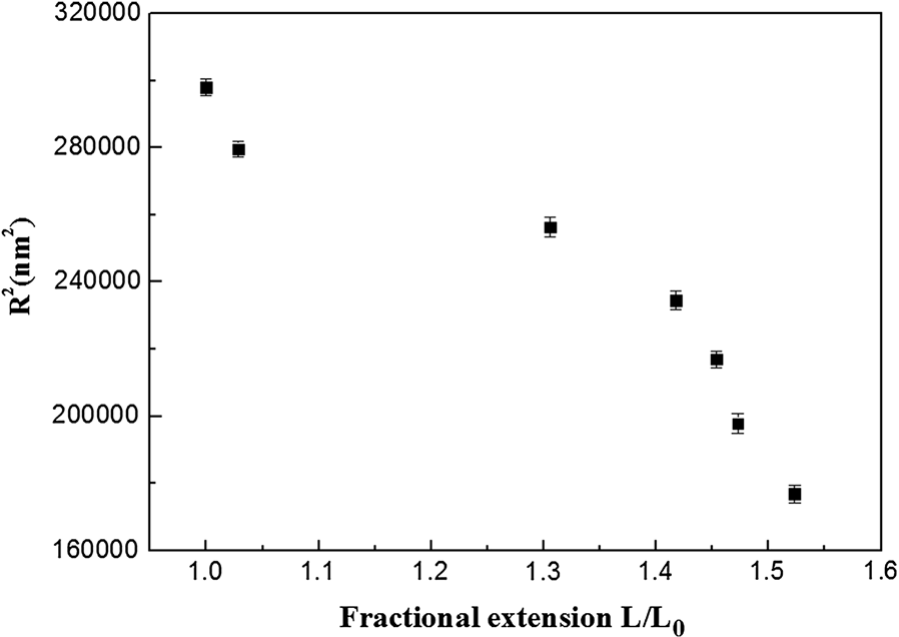
The ⟨R^2^⟩ as a function of fractional extension L/L_0_

The effect of the level of ruthenium intercalation with DNA molecule on the *L*_*p*_ and ⟨R^2^⟩ were plotted in Figs. 5, 6. The persistence length decreased with the fractional extension (L/L_0_) which was consistent with the recent results obtained by Maaloum et al. [25]. The measured value of the persistence length of dsDNA in Ru(bpy)_2_dppz^2+^ free solution was *L*_*p*_ = 54 ± 1.3 nm. This value was in good agreement with the expected value obtained from single molecule stretching experiments under physiological conditions [22]. A large decrease in the persistence length was observed on increasing the fraction extension L/L_0_ from 1.03 to 1.31 (corresponding to the ratio of Ru/bps from 0.1 to 0.2). The obvious change of the persistence length was mainly induced by the ruthenium intercalation, and the decrease of persistence length indicated the increase in dsDNA flexibility. But the plot of Fig. 6 suggested that the value of ⟨R^2^⟩ decreased with the increasing the single DNA molecule length under different ruthenium concentration. It would be speculated that the DNA helix might be bent due to ruthenium intercalation. However, by combing the increase of relative elongation of dsDNA (Fig. 4) when the ruthenium intercalation reached saturation, it would be concluded that ruthenium intercalation induced the local deformation of DNA duplex. Specifically, the DNA helix might detwisting along the axis of DNA helix at a certain degree, but we could not find the direct evidence due to limit of AFM resolution [9].

Finally, by comparing the best fitted results with the equation of McGhee and Von Hippel, the value of p was found to be 2.87, which simply meant that the saturated intercalation of Ru(bpy)_2_dppz^2+^ occurred after every 3 base pairs. In other words, the persistence length would change in a small range when the intercalation reached saturation. This was in good agreement with the persistence length results in Ru/bps solution containing ratio of Ru(bpy)_2_dppz^2+^ from 1 to 3, where the ruthenium intercalation reached saturation and the persistence length decreased to approximately 20 nm.

### Thermal dynamic stability of dsDNA measurement with HRM method under different ratios of Ru/DNAbps

**Fig. 7:**
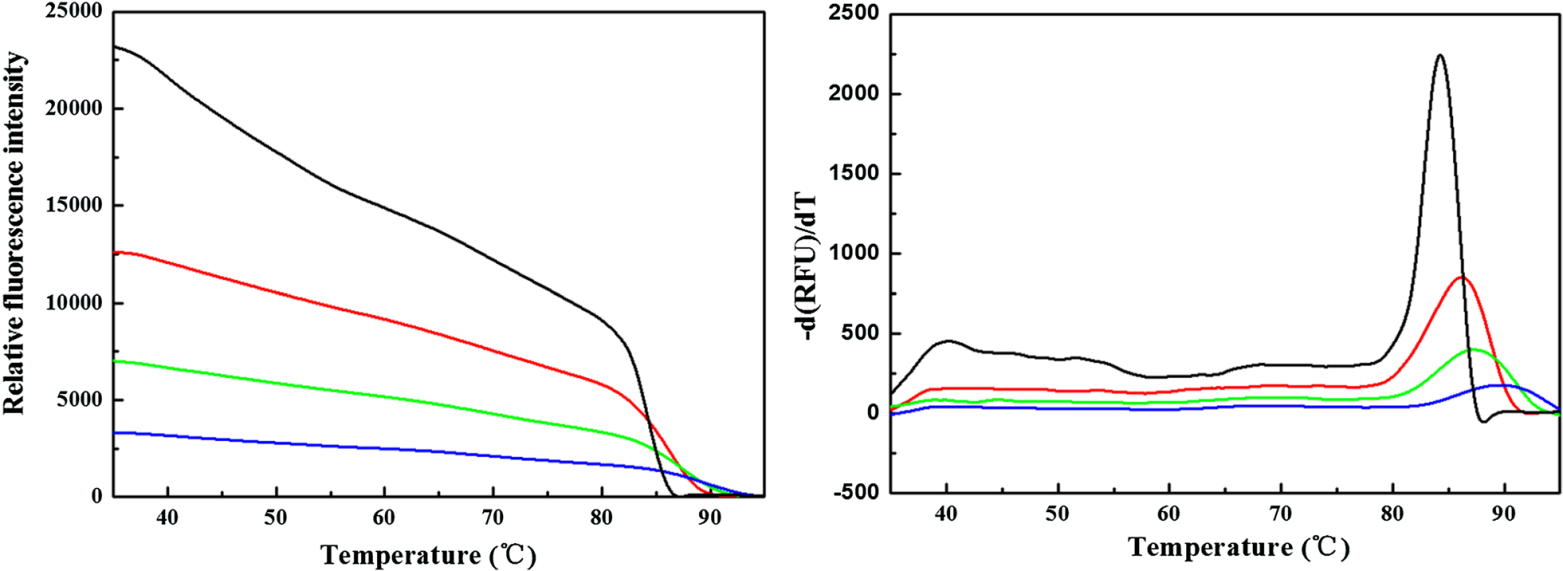
Florescence melting curves (left) and differential curves (right) of DNA duplexes with different ratios of Ru/DNAbps with ratios 0 (black), 0.1 (red), 0.2 (green), 0.33 (blue). The ratios of 1, 2, and 3 were not plotted because of the very weak fluorescence

**Fig. 8:**
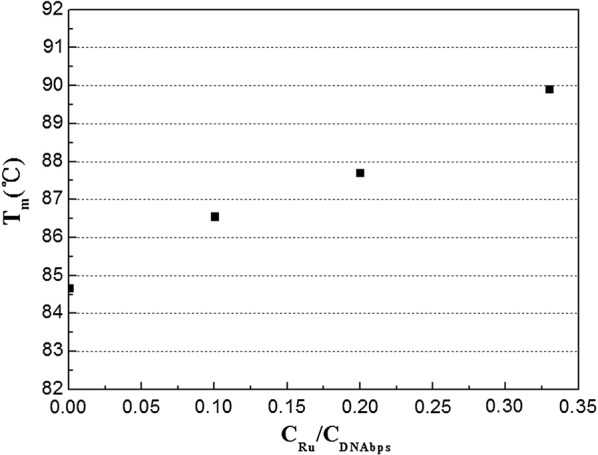
T_m_ results of dsDNA under different ratios of Ru/DNAbps

In order to investigate the thermal dynamic stability of dsDNA after ruthenium intercalation, the high resolution melting method was employed to detect the change of melting point of dsDNA as ruthenium intercalated into the duplex DNA which is a robust technique for detecting the thermal dynamic stability of dsDNA even if there is a tiny change in the structure of DNA helix [34, 35]. The melting profiles of dsDNA are shown in Fig. 7 (left) under different ratios of Ru/DNAbps. The initial relative fluorescence intensity of 80 bp duplexes was 23,000 which decreased on increasing the ratio of Ru/DNAbps because more and more ruthenium complex intercalated into DNA helix which could not be stained by the EvaGreen. The melting points of dsDNA were obtained from the differential curves and are shown in Fig. 8. The melting point of dsDNA without ruthenium compound was found to be 84.7 °C. The T_m_ values significantly increased from 84.7 to 89.9 °C with increasing the ratio of Ru/DNAbps, which could be interpreted that the thermal dynamic stability of DNA strand increased due to stacking of planar dppz ligand with DNA base pairs, but that may not disturb the integral structure of double helix. In other words, the increase of T_m_ was due to the increase of the interaction area between consecutive base pairs, which resulted in the increase of free energy [22].

## Conclusions

In present study, we used AFM and HRM methods to carefully measure the change of persistence length and thermal dynamic stability of DNA helix by changing the molar ratios of ruthenium compound to DNA base pairs. The persistence length of DNA in our study decreased from 54 to 20 nm and the melting point of dsDNA increased significantly from 84.7 to 89.9 °C after Ru(bpy)_2_dppz^2+^ intercalation. Based on these two kinds of measurement results, we speculated the obvious decrease of the persistence length due to that the intercalation of ruthenium complex partially lead to detwisting along the axis of DNA helix (local deformation) and bending of DNA helix. The decrease of persistence length also suggested that the flexibility of DNA strand increased after ruthenium intercalation from the point view of the mechanical properties of materials, which agreed well with results reported by Williams et al. [22]. In addition, the ruthenium intercalation increased the thermal dynamic stability by increasing the interaction area between two consecutive base pairs. These two kinds of results obtained with AFM and HRM can be very helpful to design the new ruthenium complex for anti-cancer drug development.

## Additional file

**Additional file 1.** The histograms of contour lengths under different ratios of Ruthenium compound to dsDNA. The fluorescence intensity of ruthenium compound at different temperatures.

## Data Availability

The datasets used or analyzed during the current study are available from the corresponding author on reasonable request.

## Abbreviations

AFM: atomic force microscopy
HRM: high resolution melting
DNA: deoxyribose nucleic acid
dsDNA: double-stand DNA
K_a_: binding constant
p: binding site size
DNAbps: DNA base pairs
Ru/DNAbps: Ru(bpy)_2_dppz^2+^ to DNA base pairs
C_Ru_: the concentration of ruthenium
C_DNAbps_: the concentration of DNA base pairs
ν: the ratio between the bound intercalator and the total concentration of DNAbps
WLC: worm-like chain
2D: two dimension
⟨R^2^⟩: the root mean square of end-to-end distance
*L*_*P*_: the persistence length
*L*_*C*_: the contour length
T_m_: melting temperature
