# Supplementary material for: Flexibility and thermal dynamic stability increase of dsDNA induced by Ru(bpy)2dppz2+ based on AFM and HRM technique
Source: BMC Chem. 2019 May 17;13(1):68. doi: 10.1186/s13065-019-0584-9 (PMC6661754; doi:10.1186/s13065-019-0584-9)
Supplement: Supplementary file 1 — Additional file 1. The histograms of contour lengths under different ratios of Ruthenium compound to dsDNA. The fluorescence intensity of ruthenium compound at different temperatures. [file 13065_2019_584_MOESM1_ESM.doc]

**Additional file 1**


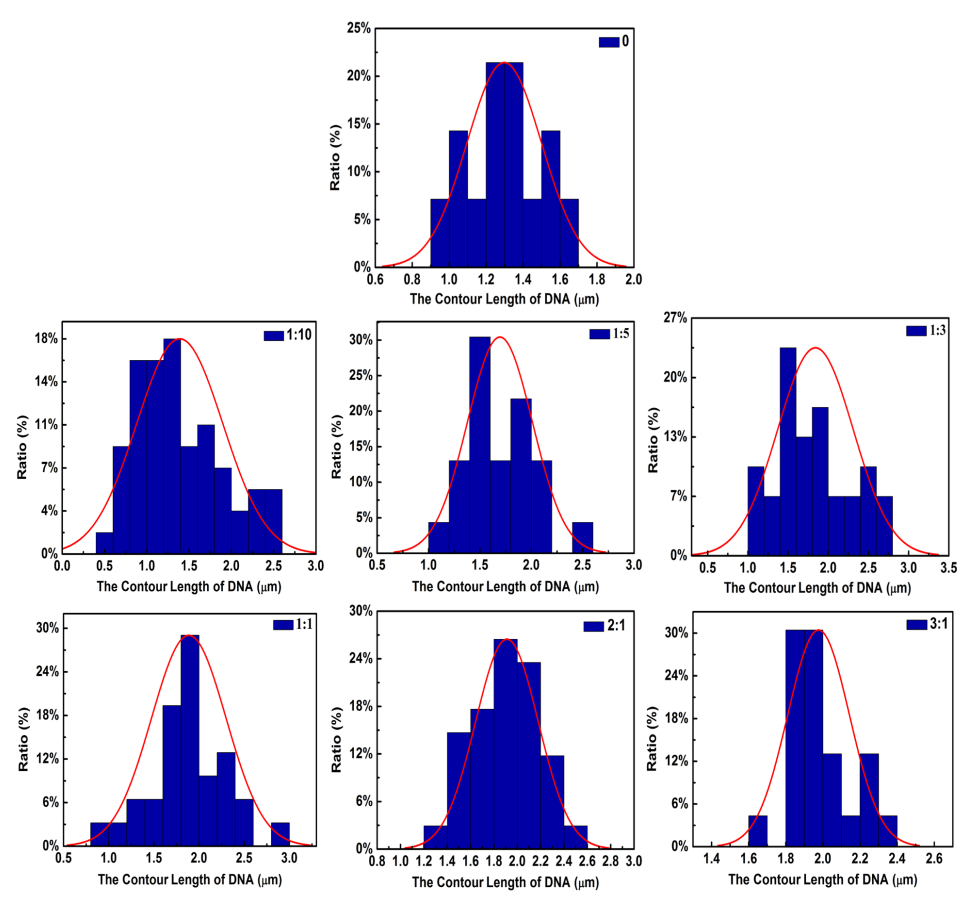

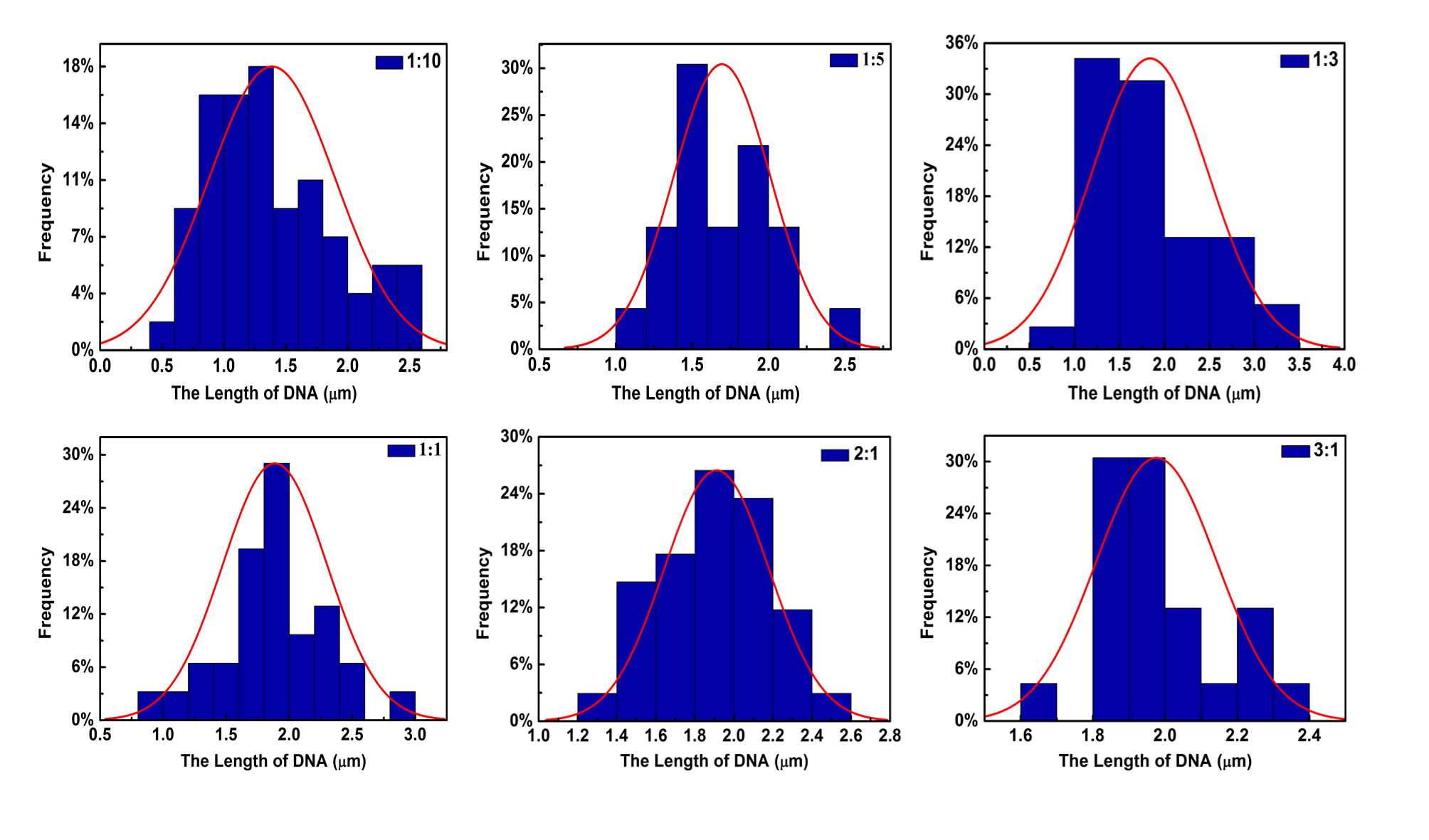


**Fig S1.** The histograms of contour lengths under different ratios of Ruthenium compound to dsDNA.


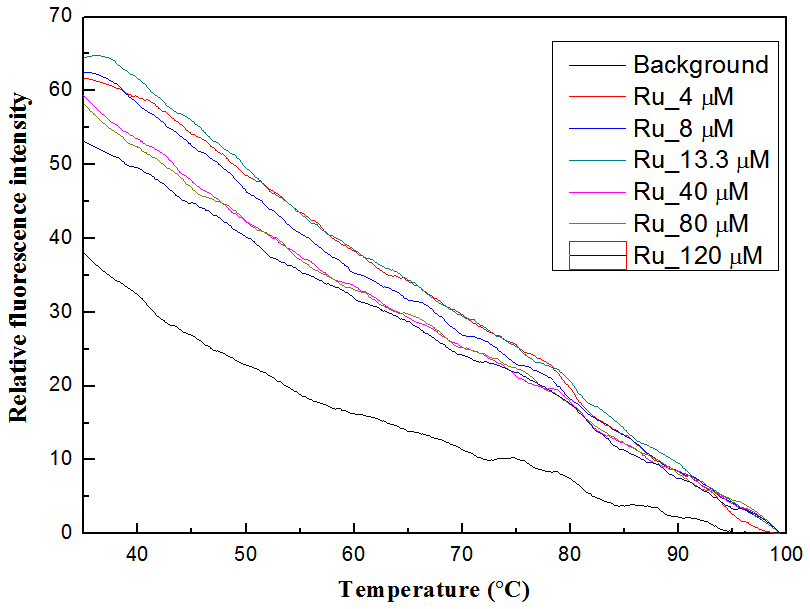


**Fig S2.** The fluorescence of ruthenium compound at different temperatures.
